# ER Stress-Inducible Factor CHOP Affects the Expression of Hepcidin by Modulating C/EBPalpha Activity

**DOI:** 10.1371/journal.pone.0006618

**Published:** 2009-08-12

**Authors:** Susana J. Oliveira, Jorge P. Pinto, Gonçalo Picarote, Vera M. Costa, Félix Carvalho, Maria Rangel, Maria de Sousa, Sérgio F. de Almeida

**Affiliations:** 1 Iron genes and the Immune System, Instituto de Biologia Molecular e Celular, Universidade do Porto, Porto, Portugal; 2 Instituto de Ciências Biomédicas de Abel Salazar, Porto, Portugal; 3 REQUIMTE, Toxicology Department, Faculdade de Farmácia, Universidade do Porto, Porto, Portugal; 4 REQUIMTE, Instituto de Ciências Biomédicas de Abel Salazar, Universidade do Porto, Porto, Portugal; Texas A&M University, United States of America

## Abstract

Endoplasmic reticulum (ER) stress induces a complex network of pathways collectively termed the unfolded protein response (UPR). The clarification of these pathways has linked the UPR to the regulation of several physiological processes. However, its crosstalk with cellular iron metabolism remains unclear, which prompted us to examine whether an UPR affects the expression of relevant iron-related genes. For that purpose, the HepG2 cell line was used as model and the UPR was activated by dithiothreitol (DTT) and homocysteine (Hcys). Here, we report that hepcidin, a liver secreted hormone that shepherds iron homeostasis, exhibits a biphasic pattern of expression following UPR activation: its levels decreased in an early stage and increased with the maintenance of the stress response. Furthermore, we show that immediately after stressing the ER, the stress-inducible transcription factor CHOP depletes C/EBPα protein pool, which may in turn impact on the activation of hepcidin transcription. In the later period of the UPR, CHOP levels decreased progressively, enhancing C/EBPα-binding to the hepcidin promoter. In addition, analysis of ferroportin and ferritin H revealed that the transcript levels of these iron-genes are increased by the UPR signaling pathways. Taken together, our findings suggest that the UPR can have a broad impact on the maintenance of cellular iron homeostasis.

## Introduction

The endoplasmic reticulum (ER) has evolved a high degree of plasticity, allowing the adjustment of its environment according to the transit of client proteins. The organelle homeostasis, however, can be threatened by numerous stimuli which overall contribute to the luminal accumulation of improperly folded proteins [reviewed in 1]. Aiming at relieving such stressful condition, a finely coordinated signaling program known as Unfolded Protein Response (UPR) is elicited [1]. Its mechanisms of action can be summarized as follows: global repression of protein synthesis; induction of ER chaperones and foldases to meet the increased folding demands and enhancement of ER-associated degradation (ERAD) of irreversibly unfolded proteins [1], [2]. The UPR employs three ER-resident transmembrane proteins that operate as proximal sensors and define independent signaling pathways towards the cytosol/nucleus: PERK (double-stranded RNA-dependent protein kinase-like ER kinase), IRE1 (inositol-requiring enzyme 1) and ATF6 (activating transcription factor 6). The output of these cascades entails the selective activation of transcription factors whose main gene targets code for components of the ER protein-processing machinery [3]. Prominent among this category is immunoglobulin heavy chain-binding protein (BiP), an ER chaperone with key sentinel activity [4].

The scope of the UPR-derived transcriptional signals goes beyond the classical targets. A paradigmatic example is cyclic AMP-responsive element binding protein H (CREBH) which, albeit activated along the UPR, executes its transcriptional activity over genes encoding inflammatory proteins [5]. Likewise, the circulating iron-transport protein transferrin (TF) was identified as a downstream target of CCAAT/enhancer-binding protein (C/EBP) homologous protein (CHOP) [6], a stress-inducible transcription factor. CHOP belongs to the C/EBP family and can heterodimerize with other members of the same class [7]. Acting as dominant negative inhibitor of other C/EBP isoforms, namely C/EBPα, CHOP was reported to down-modulate *TF* gene expression [6]. Interestingly, C/EBPα has also been described as transcriptional activator of hepcidin [8]. Although not formally tested, an identical mechanism to that depicted for *TF* was proposed to justify the impaired hepcidin transcription observed in two models of hepatic iron overload, induced by either hepatitis C virus [9] or alcohol [10].

As a major orchestrator of iron homeostasis [11], hepcidin binds to the iron exporter ferroportin and negatively regulates cellular iron release into circulation [12]. A poor induction of hepcidin despite the systemic iron overload has been found in Hereditary Hemochromatosis (HH) [13]. The leading cause of this disorder – the C282Y mutation of HFE protein [14] – was recently coupled to the activation of an UPR [15], which reinforces the interest of exploring the UPR signaling/iron metabolism interplay.

To clarify this putative interconnection, we examined whether activation of an UPR affects the expression of relevant iron-related genes. Being the cellular “factory of iron-proteins” [reviewed in 16], hepatocytes emerged as the most relevant platform for our study, herein recapitulated by the well-characterized human hepatoma HepG2 cell line. Dithiothreitol (DTT) and homocysteine (Hcys) were used as UPR inducers. Both agents interfere with disulphide bond formation, thereby burdening the ER lumen with misfolded proteins [17], [18], [19]. Using this approach, we show that the gene profiles of hepcidin, ferroportin and ferritin H are modulated throughout an active UPR. In addition, evidence supporting the involvement of C/EBPα and CHOP on the expression pattern exhibited by hepcidin is also provided.

## Results

### Experimental model of ER stress

#### Dose-response assays

**Figure 1:**
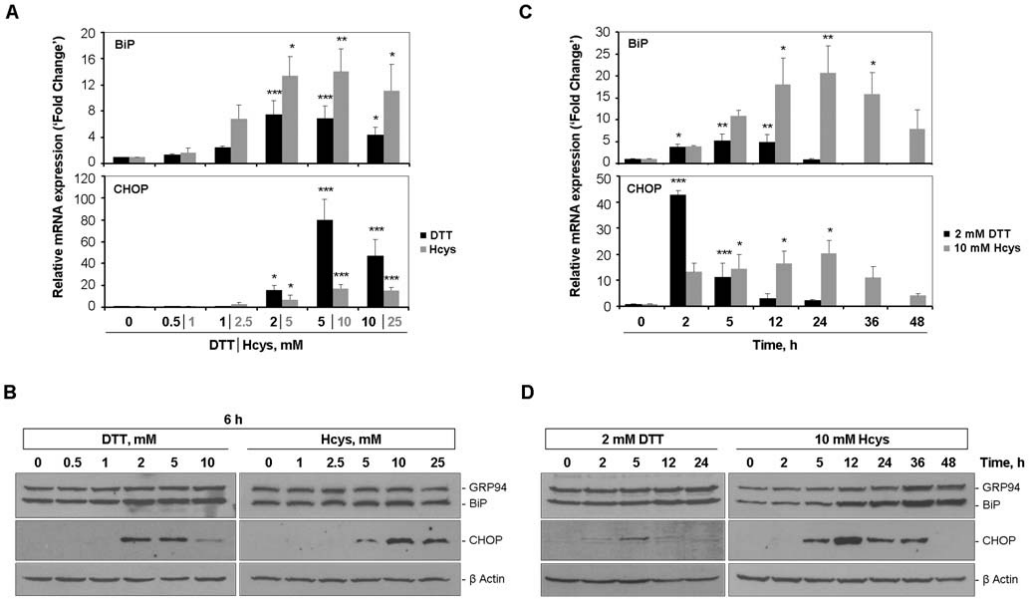
Monitoring of UPR activation markers in HepG2 cells during the DTT- and Hcys-induced ER-stress. Dose-response assays (A and B): HepG2 cells were incubated for 6 h with increasing concentrations of DTT or Hcys, as indicated. Control cells received vehicle alone. Time-course assays (C and D): HepG2 cells were exposed to 2 mM DTT or 10 mM Hcys for the indicated times. Control cells were left untreated. *A* *and* *C*, BiP and CHOP mRNA levels were assessed by real-time RT-PCR and normalized to *GAPDH* expression. Results are expressed as fold change over control-treated cells and represent the average+SD of three independent experiments. *B* *and* *D*, GRP94, BiP (using an anti-KDEL antibody) and CHOP were measured by western blot in whole cell lysates of HepG2 cells. As loading control, membranes were stripped and reprobed for β-actin. Representative blots of three independent experiments are shown. *p<0.05, **p<0.01, ***p<0.001 *vs* control.

The first part of the work was assigned to establish the minimum concentration of stressor agent able to robustly trigger an UPR. For this purpose, HepG2 cells were exposed for 6 h to increasing doses of DTT and Hcys, ranging from 0.5 to 10 mM and from 1 to 25 mM, respectively. The mRNA and/or protein levels of the ER-resident chaperones glucose regulated protein 94 (GRP94) and BiP, and of the stress-inducible transcription factor CHOP were examined as markers of UPR activation. Albeit with different magnitudes, both DTT and Hcys elicited a dose-dependent up-regulation of GRP94, BiP and CHOP, noticeable in terms of gene and protein expression (Fig. 1A and B). Of note, CHOP transcript levels were very low in basal conditions (Fig. 1A) and the respective protein was only detectable after treatment with the highest concentrations tested (DTT≥2 mM and Hcys≥5 mM; Fig. 1B). Since 2 mM DTT and 10 mM Hcys were enough to ensure a fully-activated UPR, distinguished by the simultaneous induction of BiP and CHOP, they were chosen as working concentrations for the time-course assays.

#### Time-course assays

In response to DTT, BiP and CHOP exhibited markedly different expression profiles within the 24 h time-frame analyzed. BiP transcripts increased consistently throughout the experiment, only declining in the final period of treatment, which is likely a consequence of the DTT labile properties (Fig. 1C). At the protein level, however, a persistent up-regulation of BiP was found at 24 h (Fig. 1D). Conversely, CHOP transcripts manifested a strong induction after 2 h of DTT incubation, rapidly abolished over time (Fig. 1C, black bars). This pattern of CHOP expression was further confirmed by the immunoblot results showing a protein peak at 5 h (Fig. 1D). Using Hcys as ER-stressor, BiP and CHOP mRNA levels were both gradually up-regulated over the 24 h treatment of HepG2 cells (Fig. 1C). Extending the temporal window of the assay, we observed a decline in the transcript abundance of these UPR targets until 48 h (Fig. 1C). The similarity in terms of mRNA expression was not mirrored by the protein profiles, however. As Fig. 1D illustrates, whereas BiP increased from 12 to 48 h of Hcys exposure, induction of CHOP protein became detectable at 5 h and persisted until 36 h of incubation.

### Expression of iron-related genes is modulated in the context of an active UPR

To ascertain whether triggering of an UPR had an impact on cellular iron metabolism, gene expression profiling of hepcidin, ferritin H and ferroportin was performed on HepG2 cells. The choice of these genes was based on their well documented roles in iron homeostasis [reviewed in 20].

**Figure 2:**
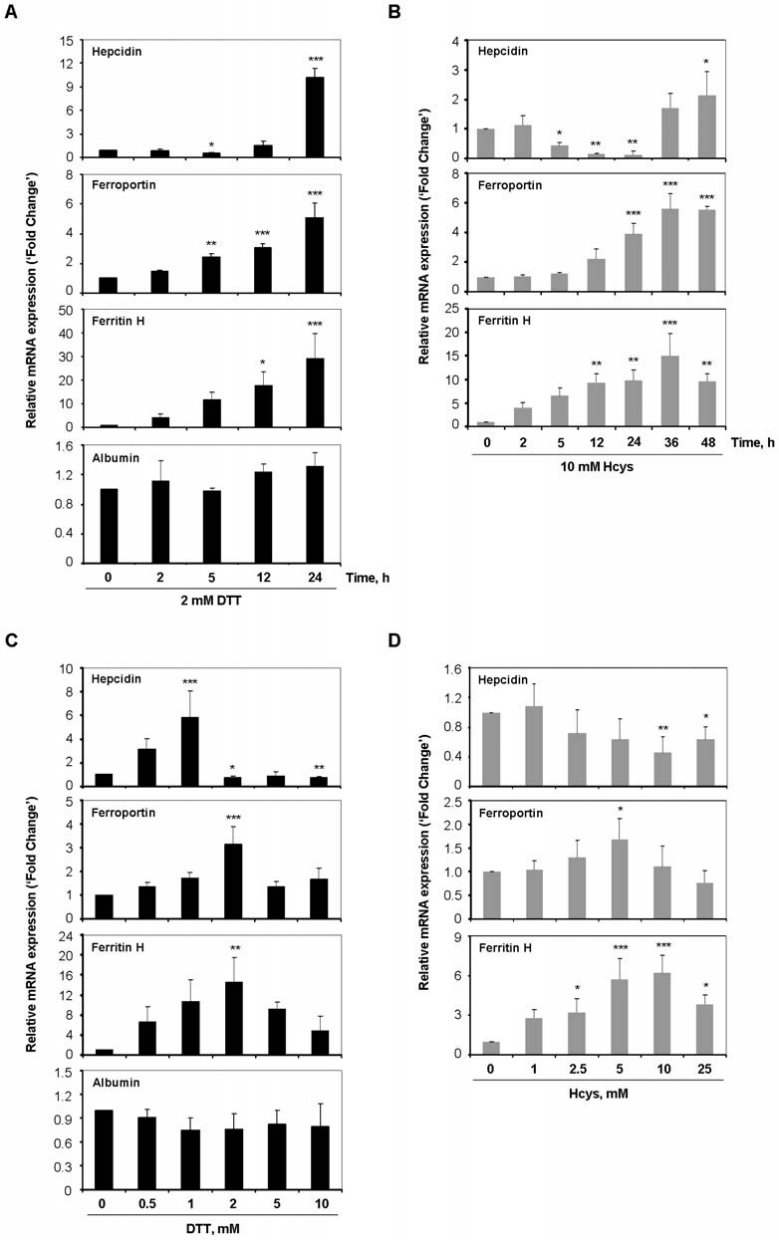
Expression of iron-related genes is modulated following UPR activation. HepG2 cells were cultured in the presence of 2 mM DTT (A) or 10 mM Hcys (B) for the indicated intervals. Untreated cells were used as control. In dose-response assays, HepG2 cells were treated for 6 h with DTT (C) or Hcys (D) at concentrations ranging from 0.5 to 10 mM and from 1 to 25 mM, respectively. Control cells were incubated with vehicle. After treatments, total RNA was isolated and mRNA levels of the “iron-genes” hepcidin, ferroportin and ferritin H assessed by real-time RT-PCR. Expression of albumin, a non-iron-related gene, was evaluated as control in cells subjected to the DTT-induced ER stress. Data were normalized to *GAPDH* and are expressed as fold change over control-treated cells. Each bar displays the average+SD of three independent experiments. *p<0.05, **p<0.01, ***p<0.001 *vs* control.

Upon 5 h of DTT treatment, the expression levels of hepcidin in HepG2 cells were significantly reduced to approximately one-half the control values, which was followed by a 10-fold up-regulation at 24 h (Fig. 2A). Nearly identical effects were produced when Hcys was used as stressor agent, although with less pronounced increase (2-fold) of hepcidin trancripts in the late stage of treatment (Fig. 2B). Ferroportin and ferritin H displayed analogous expression patterns in the presence of 2 mM DTT and 10 mM Hcys, with both mRNAs being induced in a time-dependent manner (Fig. 2A and B).

To gain additional insight into the modulation of the iron-genes under scrutiny, we assessed the influence of varying doses of each UPR inducer on their transcript levels. As Fig. 2C depicts, hepcidin mRNA showed a biphasic pattern of expression in response to DTT. Indeed, low concentrations of this stressor (0.5 and 1 mM) significantly increased hepcidin transcript levels, whereas higher doses counteracted this effect. Unlike DTT, the up-regulation of hepcidin mRNA was not evident when cells were cultured in the presence of low concentrations of Hcys. However, the down-modulation associated with higher doses of stress was maintained (Fig. 2D). Concerning the other iron-related genes, both ferroportin and ferritin H mRNAs reached a peak with 2 mM DTT, decreasing thereafter. Equivalent outcome was found in Hcys treatments, with ferroportin and ferritin H increasing in a dose-dependent fashion until 5 and 10 mM of stressor agent, respectively.

Expression levels of albumin, herein employed as control gene, were examined on cells exposed to DTT, with no differences detected in either time- or dose-dependent assays (Fig. 2A and C).

### Modulation of hepcidin expression upon DTT-elicited UPR is chelatable iron-independent

The central role of hepcidin in iron homeostasis, coupled to its marked modulation in HepG2 cells undergoing ER stress, prompted us to focus on this molecule. Therefore, the subsequent experiments were designed to dissect the regulatory mechanisms underlying hepcidin expression pattern in the presence of an active UPR.

**Figure 3:**
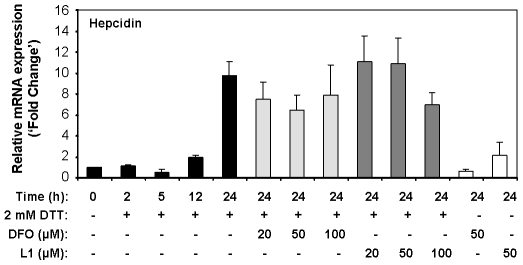
Modulation of hepcidin mRNA expression by DTT is independent of chelatable-iron. HepG2 cells were cultured in the presence of 2 mM DTT and harvested at the indicated times. For the iron chelation studies, cells were co-incubated with 20, 50 or 100 µM of DFO or L1 per culture plate. As control, cells were exposed to 50 µM of each iron chelator alone. After treatments, cDNA was synthesized from total RNA and mRNA expression levels of hepcidin were quantified by real-time RT-PCR and normalized to *GAPDH*. Data are displayed as fold change over non-treated cells and represent the average+SD of three independent experiments.

Although DTT has proven to be a useful UPR inducer [17], being a reducing agent its spectrum of action is broad and relatively unspecific. Hence, we hypothesized that DTT could accelerate Fe^3+^ reduction, thereby modulating cellular iron traffic and, consequently, regulating the expression of the genes under study [reviewed in 20]. To assess the contribution of iron to the differential expression of hepcidin in response to DTT, a kinetic analysis of HepG2 cells exposed to this drug was conducted in the presence of two distinct iron chelators: desferrioxamine (DFO) and deferiprone (L1). As displayed in Fig. 3, the 24 h up-regulation of hepcidin transcripts was preserved in the co-incubation assays, ruling-out the contribution of chelatable iron to our observations. As an additional control, BiP and CHOP expression levels were measured upon 24 h of combined treatment, with no significant differences found at this point (data not shown).

### C/EBPα and CHOP mediate the early down-modulation of hepcidin upon UPR induction

**Figure 4:**
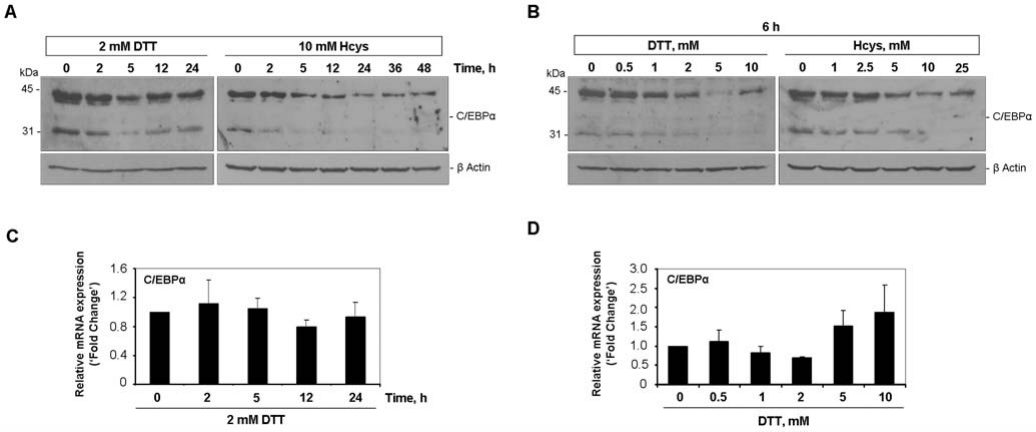
C/EBPα protein but not mRNA levels are modulated by the UPR. HepG2 cells were exposed to 2 mM DTT (A and C) or 10 mM Hcys (A) for different time periods. Control cells were left untreated. Increasing concentrations of DTT (B and D) or Hcys (B) were used to treat HepG2 cells during 6 h. Control cells were incubated with vehicle alone. *A* *and* *B*, After cell lysis, C/EBPα protein content was examined in whole extracts by immunoblot. Two forms of C/EBPα with the expected sizes (42 and 30 KDa) were detected. Molecular weights are indicated in the left side of the panels. To confirm equal lane loading β-actin was measured. Representative blots of three independent experiments are shown. *C* *and* *D*, Quantification of C/EBPα mRNA levels in DTT-treated HepG2 cells was performed by real-time RT-PCR. Data were normalized to *GAPDH* as endogenous control. Fold change relative to control cells was calculated and is displayed as the average+SD of three independent experiments.

The liver-enriched nuclear factor C/EBPα has been implicated in both up- [8] and down- [21] regulation of hepcidin in a variety of contexts. As a first step to delineate the molecular mechanism(s) involved in the UPR-dependent modulation of hepcidin expression, the levels of this transcription factor were evaluated during the time- and dose-response assays. Western blot analysis of HepG2 cells exposed to the stressor agents allowed the detection of two products with the expected sizes of 42 and 30 KDa (Fig. 4A and B), corresponding to the two C/EBPα isoforms. The lowest protein levels of C/EBPα (one half the control values) were observed after 5 h of DTT treatment (Fig. 4A), which coincides with the decreased expression previously found for hepcidin (Fig. 2A). Hence, we hypothesized that reduced amounts of C/EBPα protein might determine a poorer stimulation of the hepcidin promoter, therefore accounting for the down-modulation of its transcript levels at this time-point. The mRNA levels of C/EBPα remained unaffected over the time-course assays (Fig. 4C), suggesting that the 5 h-repression of this nuclear factor is post-translationally determined. Since CHOP protein levels also peaked 5 h post-stimulation with DTT (Fig. 1D), the possibility of CHOP participating in the modulation of C/EBPα protein described above was considered. This hypothesis was reinforced by the dose-response experiments, in which the same range of DTT concentrations (2–10 mM) accommodated both induction of CHOP (Fig. 1B) and down-modulation of C/EBPα (Fig. 4B) proteins. Once again, C/EBPα protein changes did not mirror those of the correspondent mRNA (Fig. 4D). Employing Hcys as ER-stress activator, a similar set of results was obtained. In terms of kinetics, the down-modulation of hepcidin initiated after 5 h of treatment (Fig. 2B) also overlapped with both induction of CHOP (Fig. 1D) and reduction of C/EBPα (Fig. 4A) proteins. Overall, data provided by the dose-response experiments with Hcys (Fig. 1B and 4D) were in close agreement with the C/EBPα-CHOP interplay suggested by the DTT assays.

**Figure 5:**
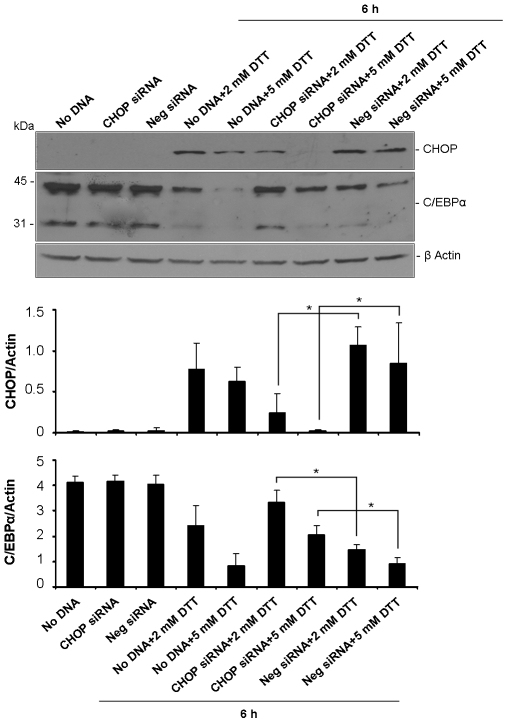
Reduction of C/EBPα protein levels depends on CHOP up-regulation. HepG2 cells were transfected with CHOP or scrambled (Neg) siRNAs for 24 h or left untreated (No DNA). DTT was added at the indicated dosage in the last 6 h of the transfection period. Efficacy of CHOP silencing was assessed in total cell lysates by western blot with anti-CHOP antibody (upper panel). Blots were also probed to C/EBPα and the two expected products (42 and 30 KDa) were detected (middle panel). β-actin was employed as loading control. The intensity of bands relative to CHOP and the 42 KDa-form of C/EBPα was quantified by densitometry, normalized to β-actin and graphically represented as average+SD of three independent experiments. *p<0.05.

To further test the hypothesis of a C/EBPα protein repression dependent on CHOP induction we used the DTT model of UPR activation. HepG2 cells were transfected with siRNA oligonucleotides targeting CHOP prior to supplementation of 2 and 5 mM DTT. Confirming the silencing efficiency, transfection of HepG2 cells with CHOP siRNAs impaired the DTT-stimulation of CHOP (Fig. 5). Importantly, down-modulation of C/EBPα protein in response to DTT (2 and 5 mM) was significantly prevented, thus supporting our initial hypothesis.

### Differential C/EBPα binding to hepcidin promoter mediates the late up-regulation of hepcidin by the UPR

**Figure 6:**
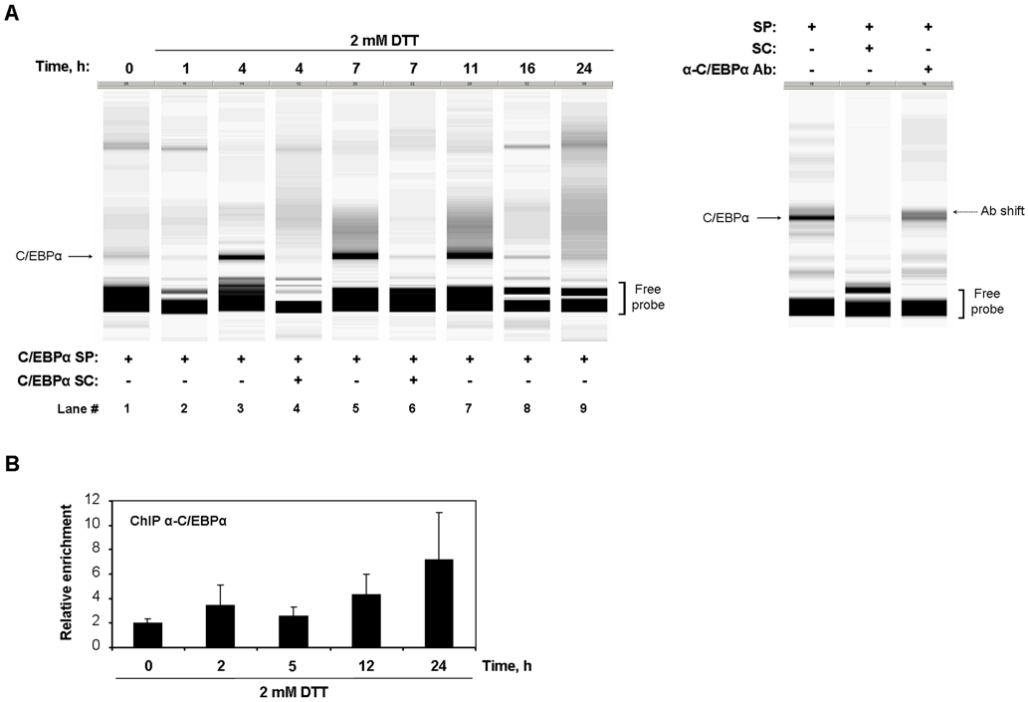
DNA-binding activity of C/EBPα is modulated over the time-course of UPR activation. HepG2 cells cultured in the presence of 2 mM DTT for the indicated intervals were subjected to fEMSA (A) and ChIP (B) analyses. *A*, Nuclear protein extracts from DTT- or control-treated cells were combined with the Cy5-labeled specific probe containing the C/EBPα consensus binding site (C/EBPα SP) and electrophoresed under native conditions. For competition assays, a 50-fold molar excess of unlabeled probe (C/EBPα SC) was used (lanes 4 and 6). The specific DNA-protein complex is indicated by an arrow. Free-probe is also shown in the bottom of the gel. The fEMSA gel view displayed is representative of four independent experiments (left panel). A specific antibody against C/EBPα was added for the supershift reaction (right panel). The partial shift is highlighted in the right side of the figure. *B*, Cross-linked chromatin was immunoprecipitated with anti-C/EBPα or anti-IgG (serum control) antibodies. The recovered chromatin samples were analyzed by quantitative real-time PCR with primers flanking the C/EBPα binding site found within the −136/+9 region of human *HAMP* promoter. Results depict the enrichment relative to serum control immunoprecipitation normalized to ChIP input values and are expressed as average+SD of three independent experiments.

Maintenance of DTT- and Hcys-induced UPR for longer than 5 or 24 h respectively, was accompanied by an up-regulation of hepcidin expression (Fig. 2A and B). Since this stimulation of hepcidin could not be directly attributed to quantitative changes in the C/EBPα protein pool (Fig. 4A), we asked whether in this phase the UPR would be modulating the binding activity of C/EBPα to the hepcidin promoter. This question was addressed *in vitro* by performing a fluorescent variant of EMSA (fEMSA), in which nuclear extracts of DTT-treated HepG2 cells were combined with a DNA probe containing the consensus binding sequence recognized by C/EBPα [22]. The single retarded complex detected was efficiently competed by an excess of unlabeled probe (Fig. 6A, *lanes 4 and 6*) and partially supershifted upon addition of an anti-C/EBPα antibody (Fig. 6A, right panel), which attested its specificity. The intensity of the C/EBPα-DNA complex, reflecting the binding activity of this nuclear factor to its consensus sequence, enhanced from 4 to 11 h and returned to baseline values beyond this time-point (Fig. 6A). Although the kinetics of C/EBPα binding did not strictly match the expression profile of hepcidin, our fEMSA results suggest that modulation of C/EBPα binding to hepcidin promoter might underlie the up-regulation of its expression detected after 24 h of DTT treatment.

To better examine how DTT affected the binding of C/EBPα to the hepcidin promoter *in vivo*, ChIP analysis was carried out. As Fig. 6B shows, following 24 h of culture in the presence of this ER stressor, the recruitment of C/EBPα to the hepcidin promoter was increased, which may account for the up-regulation of hepcidin mRNA levels detected at the same time-point.

**Figure 7:**
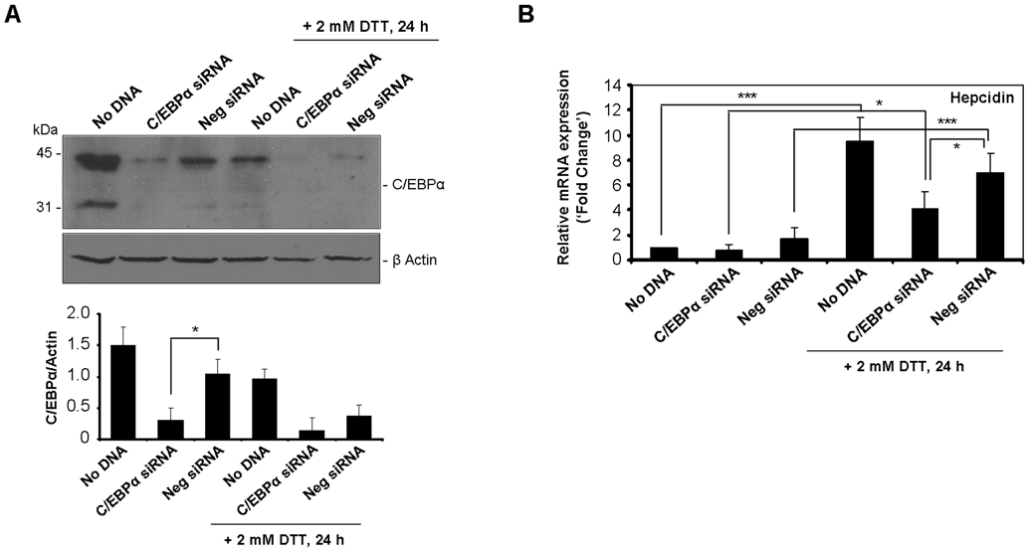
Silencing of C/EBPα partially prevents the late increase of hepcidin induced by the UPR. HepG2 cells were transfected with C/EBPα or scrambled (Neg) siRNAs for 48 h or left untreated (No DNA). Cells were exposed to DTT during the last 24 h of transfection procedure. *A*, Efficiency of C/EBPα knock-down was evaluated in whole cell extracts by western blot using an antibody against C/EBPα. The typical 42 and 30 KDa products were detected. To equalize lane loading β-actin was probed. A representative blot is shown. Intensity of the 42 KDa-band of C/EBPα was quantified by densitometry, normalized to β-actin and plotted as average+SD of three independent experiments. *B*, Total RNA of siRNA-transfected or untreated (No DNA) HepG2 was isolated. Using real-time RT-PCR, mRNA expression levels of hepcidin were measured and normalized to the *GAPDH* house-keeping gene. Data are expressed as fold change over No DNA-treated cells. *p<0.05, ***p<0.001.

To conclusively confirm the involvement of C/EBPα in the 24 h-increase of hepcidin transcript levels, a RNA interference approach was employed. Transfection of HepG2 cells with siRNA oligonucleotides against C/EBPα successfully attenuated the expression of the corresponding protein (71±20% of silencing relative to negative control siRNA-transfected cells; Fig. 7A). Moreover, C/EBPα silencing significantly abolished the responsiveness of hepcidin to the ER-stressor DTT upon 24 h of incubation (Fig. 7B), thus confirming C/EBPα as a relevant intermediary in the transcriptional regulation of hepcidin by the UPR.

## Discussion

Compelling evidence extending the UPR beyond its classical role in the mitigation of protein misfolding and proteotoxicity has been provided through its connection to obesity [23] and type 2 diabetes [24], the crosstalk with inflammation [reviewed in 25] and its impact on the cell surface expression of major histocompatibility complex (MHC) class I molecules [15], [26]. Moreover, previous reports have also suggested the existence of an interplay between the UPR and iron metabolism [6], [9], [10]. Details of this association, however, remain unclear, prompting us to exploit whether the expression of iron-related genes is influenced by ER stress-dependent mechanisms.

**Figure 8:**
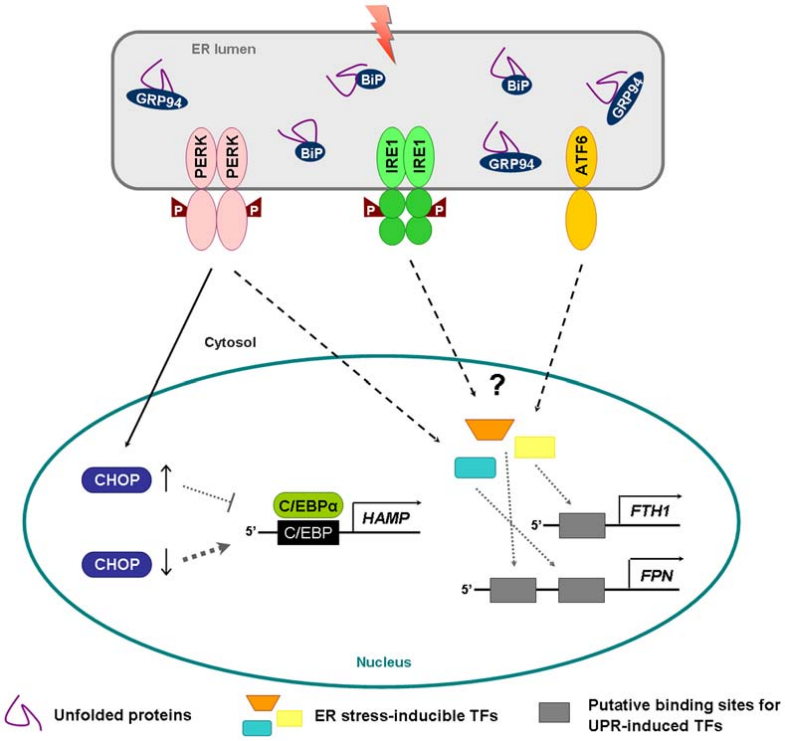
Schematic overview of the mechanisms underlying the UPR-induced modulation of iron-related genes. Activation of the PERK-dependent branch of the UPR modulates CHOP levels. This will in turn affect the C/EBPα protein pool and ultimately the stimulation of the hepcidin (*HAMP*) promoter. Regarding the other iron-genes, both ferritin H (*FTH1*) and ferroportin (*FPN*) display putative binding sites recognized by transcription factors (TFs) activated during the UPR. The presence of these regulatory elements may confer some UPR-responsiveness to ferritin H and ferroportin, increasing their expression in response to ER stress.

Hepcidin, a liver-derived peptide hormone, binds to the iron exporter ferroportin, causing its internalization and degradation, thereby blocking cellular iron efflux and intestinal iron absorption [12]. The pivotal role of hepcidin in the maintenance of systemic iron balance led us to focus on the regulation of its expression in the context of an active UPR. In our model, mRNA levels of hepcidin were significantly influenced by the UPR elicited by DTT and Hcys. In addition, the results enabled us to identify C/EBPα as an important mediator of such modulation. Our interest on this transcription factor was driven by two previous findings: i) C/EBPα is a liver-enriched nuclear factor known to regulate hepcidin transcription [8] and ii) C/EBPα can heterodimerize with other members of the same family, some of which induced by the UPR signaling program [7]. CHOP is one of these members, whose higher protein levels consistently overlapped with the down-modulation of both C/EBPα protein and hepcidin transcripts in the experimental conditions used. This observation allowed us to hypothesize a concerted regulation between CHOP and C/EBPα, successfully supported by the CHOP silencing assays (Fig. 8 depicts a schematic representation of the proposed model). According to these data, the rise of CHOP levels upon ER stress leads to a decrease of C/EBPα protein content, which may limit the availability of this nuclear factor to stimulate hepcidin transcription, with the concomitant down-modulation of its mRNA levels. An analogous interplay between C/EBPα and CHOP was previously described in pre-adipocytes, where CHOP up-regulation prevented C/EBPα expression [27], [28]. Similarly, the negative regulation of C/EBPα expression by CHOP was observed in livers from tunicamycin-challenged mice and proposed as a key mechanism linking ER stress to the disruption of hepatic lipid metabolism [29].

We found substantial differences in the time-course expression of the UPR downstream effectors. While increased protein levels of BiP were sustained, the up-regulation of CHOP was more transient. This pattern is in agreement with the concept of chronic *vs* acute stress, distinguished by detectable levels of BiP or CHOP, respectively [30]. In addition, the loss of CHOP expression is likely on the basis of hepcidin recovery observed in the long-term incubation with both stressors. Accordingly, our ChIP analysis revealed that the endogenous hepcidin promoter of HepG2 cells was enriched in the C/EBPα transcription factor after 24 h of DTT-evoked UPR. One can speculate that this occurs as an attempt by the cell to compensate for the deficit of hepcidin expression imposed by CHOP up-regulation. The modulation of C/EBPα binding activity throughout the ER-stress response was likewise evidenced by the fEMSA experiments, although with different kinetics. The discrepancy between the fEMSA and ChIP data may reflect the inability of the former method to efficiently mimic binding conditions found *in vivo*. It can also be explained by differential affinity of C/EBPα for the binding sites used in each assay: a C/EBPα consensus binding sequence in fEMSA and the native C/EBPα-responsive elements within hepcidin promoter in ChIP.

The contribution of iron to the up-regulation of hepcidin here reported was excluded when the UPR was chemically elicited by DTT under iron chelating conditions. Furthermore, it has been shown that hepatocytes in culture fail to increase hepcidin production in response to iron loading [31], [32].

Our *in vitro* model system of a pharmacologically-elicited UPR could render valuable clues when extrapolated to a physiological context. Hepcidin levels are known to be abnormally low in HFE-linked Hereditary Hemochromatosis (HH) [13]. Considering that markers of an active UPR were formerly found in individuals homozygous for the C282Y mutation of HFE [15], it is tempting to speculate that the CHOP-C/EBPα interconnection here described may contribute to the impaired induction of hepcidin expression regardless of the iron burden. The intersection between the UPR signaling pathways and the transcriptional regulation of hepcidin could therefore contribute to explaining some of the phenotypic variation amongst HH patients [33].

Regarding the other two genes examined in the present study, up-regulation of ferroportin and ferritin H was found in HepG2 following the UPR activation, both in dose- and time-response assays. Although beyond the scope of this work, some hypotheses can be drawn concerning the molecular pathways involved in such outcome (summarized in Fig. 8). Apart from the supra-mentioned post-translational regulation by hepcidin [12], expression of ferroportin is also controlled by a well established Iron Responsive Element (IRE)/Iron Regulatory Protein (IRP) mechanism [34]. Nonetheless, elevated hepatic mRNA levels of ferroportin were detected in HFE C282Y homozygous despite iron overload [13], [35], thus suggesting that transcriptional events also contribute to its regulation [36]. In line with this, *in silico* analysis of ferroportin gene and promoter showed the presence of a number of putative binding sites for transcription factors commonly induced during the UPR (e.g. ATF/CRE, AARE, CHOP-binding sequence; http://www.genomatix.de). Hence, we reasoned that the presence of such regulatory elements might confer some UPR-responsiveness to ferroportin.

Ferritin is an iron-storage protein whose regulation also depends on the IRE/IRP post-transcriptional system [37]. Interestingly, the existence of an antioxidant-responsive element (ARE) activated through binding to Nrf2, a nuclear factor induced by the UPR program [38], was reported for the H- and L- isoforms of ferritin gene [39]. Taken together, this report and our experimental data are indicative of a complementary/additive action driven by both iron excess and UPR activation towards ferritin mRNA up-regulation.

The boost of ferroportin and ferritin H can be envisaged as a mechanism to prevent intracellular accumulation of free iron. Our data of albumin expression seem to exclude a mere UPR-“side effect” as the reason behind our observations, supporting the likelihood of their physiological relevance. Whether the modulation of iron-related genes is part of a general UPR strategy aiming at maintaining cell viability and homeostasis or, instead, it represents a novel pathway to specifically control the expression of a subset of genes related to iron metabolism, remains the subject for further study.

## Materials and Methods

### Cell culture

Human hepatoma HepG2 cells were maintained in Dulbecco's modified Eagle's medium (DMEM) with GlutaMAX (Gibco, Grand Island, NY), supplemented with 10% heat-inactivated fetal bovine serum (Gibco, Grand Island, NY) and 1% penicillin/streptomycin/amphotericin (Sigma, St. Louis, MO). Cells were cultured at 37°C in a humidified atmosphere containing 5% CO_2_.

### Induction of ER stress and iron chelation

HepG2 cells were seeded at a density of 1.5×10^6^ cells in 60-mm diameter culture dishes. One day after plating, cells were exposed to dithiothreitol (DTT; Sigma-Aldrich, St. Louis, MO) or homocysteine (Hcys; Sigma-Aldrich, St. Louis, MO) as follows: 1) for dose-response assays, cells were incubated for 6 h with increasing concentrations of the ER-stressors (ranging from 0.5–10 mM or 1–25 mM for DTT and Hcys, respectively); 2) for time-course experiments, cells were cultured in the presence of 2 mM DTT from 2–24 h or 10 mM Hcys from 2–48 h. Control cells were treated with vehicle alone. Stock solutions of DTT and Hcys were prepared and frozen as single-use aliquots to maintain consistency among experiments. In the iron chelation experiments, DTT-stimulated ER stress was induced in the presence of either desferrioxamine (DFO; Novartis Pharma) or deferiprone (L1; Sigma-Aldrich, St. Louis, MO) at 20, 50 or 100 µM per culture plate. mRNA levels of target genes were assessed in cells exposed to both DTT and iron chelators following 24 h of incubation.

### RNA isolation and real-time RT-PCR

Total RNA was isolated from HepG2 cells using the RNeasy Midi Kit (QIAGEN, Hilden, Germany), followed by genomic DNA digestion with TURBO DNA-free (Ambion, Austin, TX). Reverse transcription was obtained from 1 µg of DNase-treated RNA employing the SuperScript First-Strand Synthesis System (Invitrogen, Carlsbad, CA), according to the manufacturer's guidelines. For real-time quantification of mRNA levels, the synthesized cDNA's were amplified in duplicate by PCR in an iCycler iQ5 (Bio-Rad, Hercules, CA) using iQ SYBR Green Supermix (Bio-Rad, Hercules, CA). At the end of the PCR cycling, melting curves were generated to ascertain the amplification of a single product and the absence of primer dimers. All the primers used are listed in Table 1. Results were normalized to GAPDH as endogenous control. Relative expression levels were calculated as follows: 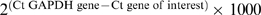.

**Table 1:** Sequences of the oligonucleotide primers used for quantitative real-time RT-PCR analysis.

Gene	Accession number	Protein coded	Forward primer (5′–3′)	Reverse Primer (5′–3′)
GRP78	M19645	BiP	CCTGGGTGGCGGAACCTTCGATGTG	CTGGACGGGCTTCATAGTAGACCGG
GADD153	S40706	CHOP	GCCTTTCTCCTTTGGGACACTGTCCAGC	CTCGGCGAGTCGCCTCTACTTCCC
HAMP	NM_021175	Hepcidin	ATGGCACTGAGCTCCCAGAT	TTCTACGTCTTGCAGCACATCC
CEBPA	NM_004364	C/EBPα	CTAGAGATCTGGCTGTGGGG	TCATAACTCCGGTCCCTCTG
SLC40A1	NM_014585	Ferroportin	CCCGGAGACAAGTCCTGAATC	TGGCCCATTGCCACAAAGGAG
FTH1	AF088851	Ferritin H	CAGAACTACCACCAGGACTCAGA	TAGCCCGAGGCTTAGCTTTCA
ALB	NM_000477	Albumin	CAAAAACATGTGTTGCTGATGA	CTTGTTTTGCACAGCAGTCAG
GAPDH	NM_002046	GADPH	GAAGGTGAAGGTCGGAGTC	GAAGATGGTGATGGGATTTC

### Antibodies and Western blot

Mouse anti-KDEL and rabbit anti-β-actin antibodies were purchased from Abcam (Cambridge, UK). Goat anti-C/EBPα (C-18) and rabbit anti-GADD153 (R-20) antibodies were supplied by Santa Cruz Biotechnology (Santa Cruz, CA). Following treatment with the ER stressors, HepG2 cells were harvested and the pellets lysed in ice-cold lysis buffer (300 mM NaCl, 50 mM Tris-HCl pH 7.4, 1% Triton X-100) supplemented with 1x Complete EDTA-free protease inhibitor cocktail (Roche Diagnostics, Mannheim, Germany). Total protein content of lysates was measured using the *RC/DC* Protein Assay (Bio-Rad, Hercules, CA) and 30 µg resolved by electrophoresis on 12% SDS-polyacrylamide gels. Proteins were then transferred to nitrocellulose Hybond-C Extra membranes (Amersham Biosciences, Little Chalfont, UK). After blocking with 5% dry milk in Tris-buffered saline containing 0.05% Tween 20 (TBS-T) for 1 h, primary antibody incubations were performed overnight at 4°C. Blots were then washed three times with TBS-T and incubated with appropriate horseradish peroxidase-conjugated secondary antibodies (Molecular Probes, Eugene, OR) for 1 h at room temperature. After washing with TBS-T, signal was developed with the SuperSignal West Pico (Pierce, Rockford, IL) chemiluminescence kit and the blots exposed to CL-X Posure films (Pierce, Rockford, IL). For normalization of protein loading, blots were stripped and reprobed with an antibody against β-actin.

### Preparation of nuclear extracts and Electrophoretic Mobility Shift Assay (EMSA)

Nuclear extracts from DTT- and control-treated cells were prepared essentially as described by Schreiber *et al.* [40]. Briefly, after harvesting and washing with ice-cold PBS, cells were suspended in hypotonic buffer (10 mM HEPES pH 7.9, 1.5 mM MgCl_2_, 10 mM KCl, 0.5 mM EDTA, 0.1 mM EGTA, 0.1% IGEPAL, 1 mM DTT, 1x Complete EDTA-free protease inhibitor cocktail [Roche Diagnostics]). Upon 15 min of incubation on ice, the nuclei were pelleted by centrifugation at 2000 *g* for 10 min and resuspended in high-salt buffer (20 mM HEPES pH 7.9, 1.5 mM MgCl_2_, 20% glycerol, 0.5 mM EDTA, 420 mM NaCl, 1 mM DTT, 1x Complete EDTA-free protease inhibitor cocktail [Roche Diagnostics]). Following incubation on ice for 30 min with gentle agitation, nuclear debris were removed by centrifugation at 16000 *g* for 25 min and the nuclear extract present on supernatants stored at −80°C. To investigate specific DNA-protein interactions, a fluorescence based EMSA was employed according to previously reported procedures [41]. The following single-stranded oligonucleotides encompassing the C/EBPα consensus sequence element [22] were purchase from Thermo Scientific (Waltham, MA): 5′-Cy5-CTAGGGCTTGCGCAATCTATATTCG-3′ (Cy5-labeled sense specific probe), 5′-CTAGGGCTTGCGCAATCTATATTCG-3′ (sense specific competitor) and 5′-CGAATATAGATTGCGCAAGCCCTAG-3′ as antisense complementary oligonucleotide. To generate double-stranded probes for EMSA, single stranded oligonucleotides were annealed by heating to 95°C for 1 min, followed by 30 min at 20°C. For binding reactions, 20 µg of nuclear extract was incubated with 1 pmol of double-stranded Cy5-labeled probe in binding buffer containing 20 mM HEPES pH 7.9, 50 mM KCl, 1 mM EDTA, 1 mM DTT, 10% glycerol and 250 ng poly[dI.dC] (Sigma, St. Louis, MO). Binding reactions were carried out overnight at 4°C and then subjected to electrophoresis through 5% nondenaturing polyacrylamide gels using an ALF-Express DNA sequencer (Amersham Pharmacia Biotech, Uppsala, Sweden). The temperature was maintained at 10°C by an ALFexpress II Cooler external thermostat (Amersham Pharmacia Biotech, Uppsala, Sweden). Signals were analyzed using ALFwin 1.03 software (Amersham Pharmacia Biotech). For the competition assays, 50-fold excess unlabeled probe was added to the reaction 1 h prior to incorporation of the labeled duplex. For supershift experiments, nuclear extracts were pre-incubated overnight at 4°C with 2 µL of antibody against C/EBPα (C-18X; Santa Cruz Biotechnology, Santa Cruz, CA) and the labelled probe added 1 h before the loading onto the gel.

### Chromatin Immunoprecipitation (ChIP)

HepG2 cells were grown in 100-mm diameter culture dishes to ∼70% of confluence and subjected to time-course assays with 2 mM DTT as depicted above. ChIP analysis was performed as previously described by Kuo *et al.* [42] with some modifications. Cells were cross-linked in 1% formaldehyde (directly added to the culture medium) for 10 min at 37°C. After washing twice with cold PBS, cells were scraped and lysed in SDS lysis buffer (1% SDS, 10 mM EDTA, 50 mM Tris-HCl pH 8.1) supplemented with 1x Complete EDTA-free protease inhibitor cocktail for 30 min on ice. Chromatin was shared into 500–1000 bp size fragments by sonicating the cell lysates with a Branson Sonifier 250 (10 cycles of 20 sec at 30% amplitude). After removing cell debris by centrifugation at 14000 *g* for 10 min, an aliquot of the supernatant was saved as input DNA. The remainder was 10-fold diluted in ChIP dilution buffer (0.01% SDS, 1.1% Triton X-100, 1.2 mM EDTA, 16.7 mM Tris-HCl pH 8.1, 167 mM NaCl) and pre-cleared with Protein A Sepharose beads (Sigma-Aldrich, St. Louis, MO) during 2 h at 4°C. Immunoprecipitation of chromatin was carried out overnight at 4°C using rabbit anti-C/EBPα antibody (14AA; Santa Cruz Biotechnology) or rabbit anti-IgG (Santa Cruz Biotechnology) as serum control. Immune complexes were recovered with Protein A Sepharose beads for 2 h at 4°C. The beads were then washed sequentially with: a) Low-salt buffer (0.1% SDS, 1% Triton X-100, 2 mM EDTA, 20 mM Tris-HCl pH 8.1, 150 mM NaCl); b) High-salt buffer (as previous but with 500 mM NaCl); c) LiCl buffer (0.25 M LiCl, 1% NP-40, 1% deoxycholic acid, 1mM EDTA, 10 mM Tris-HCl pH 8.1) and d) TE buffer (1 mM EDTA, 10 mM Tris-HCl pH 8.1). The immune complexes were eluted with elution buffer (1% SDS, 0.1 M NaHCO_3_) and protein-DNA crosslinks reversed for 6 h at 65°C in the presence of 0.2 M NaCl. Following Proteinase K (Sigma-Aldrich, St. Louis, MO) digestion for 1 h at 45°C, DNA was extracted with phenol/chloroform, ethanol precipitated and dissolved in TE buffer containing RNase H. The chromatin fragments obtained upon immunoprecipitation were analyzed by quantitative real-time PCR, using 5 µL of DNA template, iQ SYBR Green Supermix and the primers as follows: 5′-TGTCGCTCTGTTCCCGCTTATC-3′ (forward) and 5′-TCTGGTGTCTGGGACCGAGTGA-3′ (reverse). This primer set was designed to amplify the −136/+9 region of human *HAMP* promoter that contains a literature-annotated C/EBPα binding site [8]. The relative enrichment was calculated by the ΔΔCt method: upon normalization to the corresponding ChIP input, values were corrected for non-immune background according to the equation as follows 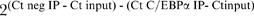.

### siRNA transfection

Small interfering RNA's (siRNA's) targeting human C/EBPα and CHOP were designed and synthesized by Eurogentec (Ougrée, Belgium). The siRNA sequences were as follows: C/EBPα (5′-CGCACCUGCAGUUCCAGAU-3′ and 5′-GAGACGUCCAUCGACAUCA-3′); CHOP (5′- GCGCAUGAAGGAGAAAGAA-3′ and 5′-GCUGAGUCAUUGCCUUUCU-3′). A scrambled siRNA (Eurogentec) was used as negative control. To improve the transfection efficiency, a cell-suspension variant of the standard protocol was applied to HepG2 cells. Briefly, after trypsinization and dilution in antibiotic-free medium, 3×10^5^ cells were transferred to 6-well plates containing the siRNA duplexes complexed with Lipofetamine 2000 (Invitrogen, Carlsbad, CA), previously diluted in Opti-MEM (Gibco, Grand Island, NY). Following 48 or 24 h of transfection, for C/EBPα or CHOP respectively, the knock-down effectiveness was evaluated by immunoblot analysis.

### Statistics

Results are expressed as average+standard deviation (SD) of three independent experiments. To detect significant differences among sample means, one-way ANOVA repeated measurements, followed by Tukey test was used. Statistical significance was considered at *p*<0.05. Statistical analysis was performed using SigmaStat software.
